# Morphological Development of the Hip in Normal Infants Under Six Months of Age by the Graf Ultrasound Method

**DOI:** 10.3389/fped.2022.914545

**Published:** 2022-05-09

**Authors:** Bing Liu, Xiaoyun Hu, Lianyong Li, Shuxi Gao

**Affiliations:** ^1^Department of Pediatric Orthopedics, Shengjing Hospital of China Medical University, Shenyang, China; ^2^Department of Ultrasound, Shengjing Hospital of China Medical University, Shenyang, China

**Keywords:** infants, hip, ultrasonography, Graf method, morphometry

## Abstract

**Objective:**

This large-sample observational study aims to analyze the morphological development of the hip joint in Chinese normal infants under 6 months of age by the Graf ultrasound method.

**Methods:**

The clinical and ultrasound data of infants who underwent early screening for developmental dysplasia of the hip (DDH) in the authors' clinic from January 2011 to December 2019 were analyzed retrospectively. The standard Graf method was used to measure the hip joint α angle, β angle and femoral head coverage (FHC). The infants with Graf type I or IIa hips were included in this study. All infants were grouped by age. FHC, α and β angles were compared among different study groups.

**Results:**

A total of 3,067 infants (6,134 hips) were included in the study. There were 1,164 males and 1,903 females with an average age of 77 days (1–180 days). The mean α angle was 62.4 ± 3.6° on the left and 63.2 ± 3.5° on the right (*P* < 0.001). The mean β angle was 55.6 ± 4.5° on the left and 54.8 ± 4.5° on the right (*P* < 0.001). The average FHC was 54.2 ± 4.6% and 54.8 ± 4.2%, accordingly (*P* < 0.001). The α angle and FHC of females was significantly smaller than that of males (*P* < 0.001). While the left β angle in females was slightly larger than males (*P* = 0.014), there were no significant differences in the right β angle between the two sexes (*P* = 0.150). During the first 3 postnatal months, the α angle and FHC increased while β angle decreased with age. However, the α and β angles and FHC were stable at a relatively constant level from the 4 to the 6^th^ postnatal month.

**Conclusions:**

The normal infant hip reveals progressive maturation during the first 3 months after birth, and then enters a plateau period during the 4 to 6^th^ month. The development of hip joint in females and of the left side slightly lags behind that in males and of the right side, which is consistent with the observation that DDH is more common in females and on the left hips.

## Introduction

Ultrasound has been widely used since the 1980's for newborn screening and diagnosis of developmental dysplasia of the hip (DDH), as well as for dynamic follow-up after treatment. Ultrasound has the advantages of being radiation-free compared to radiography. This approach can display the cartilage and soft tissue structures of the hip joint, which is particularly important in infants and children whose hip joints have not yet fully ossified. In recent years, several methods of hip ultrasonography and grading have become available. Among these approaches, the most common method is the static ultrasound method proposed by Graf (1), which measures the development of the bone and cartilage of the acetabular roof by measuring the α and β angles of the standard coronal plane of the hip joint. Based on this methodological foundation, Morin et al. (2) evaluated the coverage of the bony acetabular margin by measuring the percentage of femoral head coverage (FHC). In addition, FHC could also be used to differentiate between subluxation, frank dislocation, or unstable hips (2, 3).

The Graf method classifies the hip joint by measuring the α and β angles. The α angle, whose significance is similar to that of the acetabular index, reflects the degree of inclination of the bony acetabular roof. A smaller inclination of the acetabular roof indicates a larger α angle and better bony acetabular development, and vice versa. On the other hand, the β angle reflects the degree of development of the cartilaginous acetabular roof that is continuous with the bony acetabular rim, with the normal cartilaginous acetabular roof pointing laterally and inferiorly. Therefore, smaller β angles indicate better development of the cartilaginous acetabular roof rim, and vice versa. Together, α and β angles, and FHC provide a better assessment of the head-acetabulum relationship (2–4).

Although the α angle, β angle, and FHC are the most common parameters in the clinical assessment of hip development in infants under 6 months of age, their accuracy and reliability remain controversial (5, 6). Possible reasons may include inconsistent measurement methods or inherent differences in the degree of hip development. The development of hip morphology is a dynamic process. While infancy is the first peak of postnatal hip development, it is unclear whether there is an age-dependent changes in each of these ultrasound measurement parameters. Elucidating the growth pattern of hip morphological parameters with age in normal infants is an important foundation for diagnosing hip joint abnormalities. However, there have been few studies on the developmental patterns of hip ultrasound measurements in normal infants. The available data are mostly from Europe or North America (7, 8). Little data on Chinese infants are available (9). Therefore, the purpose of this study is to elucidate the developmental patterns of α angle, β angle, and FHC of the hip in normal Chinese infants under 6 months of age through observation of a large sample. The findings from this study are expected to provide reference data for early diagnosis, treatment, and monitoring of clinical DDH.

## Materials and Methods

### Study Design

This study was approved by the Institutional Review Board of our hospital. Clinical and hip ultrasound imaging data of infants who underwent early screening for DDH at our pediatric orthopedic clinic between January 2011 and December 2019 were retrospectively analyzed. Inclusion criteria were as follows: (i) age ≤ 6 months; (ii) clear diagnostic hip ultrasound findings; (iii) Graf hip ultrasound type I or type IIa. Exclusion criteria were as follows: (i) incomplete case data; (ii) positive family history of DDH and history of oligohydramnios and breech pregnancy; (iii) any abnormity related to musculoskeletal and joint development; (iv) clinically unstable hip (i.e., positive Barlow test); (v) history of preterm birth; (vi) initial hip ultrasound diagnosis of Graf type IIa without reversal to type I on re-examination after 3 months of age.

### Clinical Assessment of the Hip

**Figure 1:**
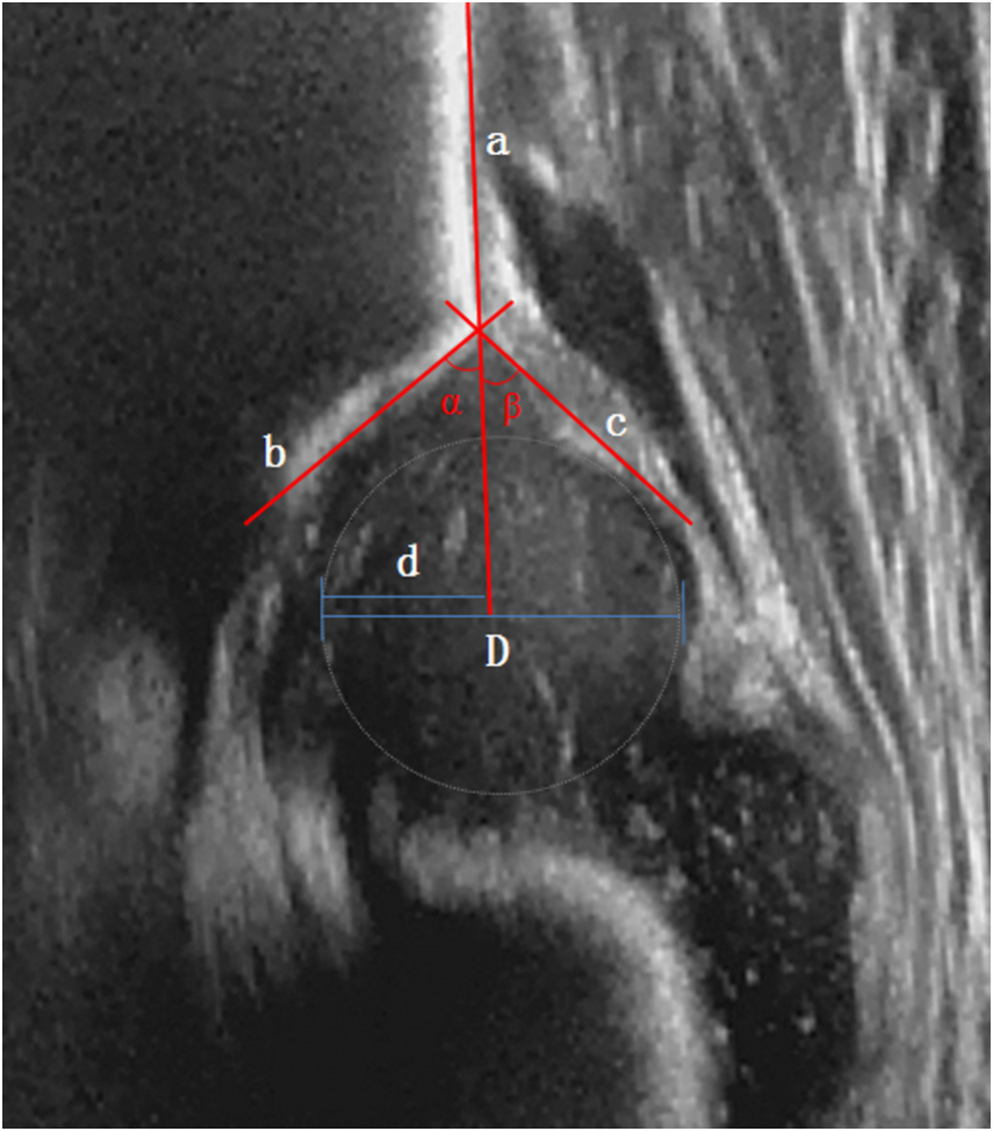
Measurements of α angle, β angle, and femoral head coverage by Graf methods.

Hip ultrasound and classifying was performed as described by the Graf method (1). The α angle, β angle, and FHC were measured. All ultrasound examinations were performed by the same senior sonographer. A line extending from the ilium was made on a standard coronal image of the hip and designated as the baseline a. Another line between the bony acetabular inflection point (the inflection point between the baseline a and the bony acetabular roof) and the most medial endpoint of the bony acetabular roof was designated line b. The line between the bony acetabular inflection point and the most lateral point of the acetabular labrum was designated line c. The angle between lines a and b is the α angle, while the angle between lines a and c is the β angle. Next, a tangent line passing through the most medial and lateral edges of the femoral head and parallel to baseline a was drawn. The bony coverage distance d of the femoral head and the maximum diameter D of the femoral head were measured, and FHC was calculated as d/D × 100% (Figure 1).

In accordance with the Graf criteria (1), the hips were classified into type I, type II (divided into subtypes a, b, c, and d), type III, or type IV based on the α and β angles. Among these types, Graf type I (α angle > 60°, β angle <55°) and IIa (<3 months, α angle 50–60°, β angle 55–77°) hips were defined as normal and included in the study. All infants were stratified in different age groups (≤30 days: 1-month group; 30–60 days: 2-month group; by parity of reasoning, and 150–180 days: 6-month group).

### Statistical Analysis

SPSS 11.5 software was used for statistical analysis. Measurement data are expressed as means ± standard deviations. The *t*-test for independent samples was used to compare the differences in α and β angles, and FHC between the sexes. The paired *t*-test was used to compare differences in α and β angles, and FHC between the right and left sides. ANOVA was used to compare the differences in α and β angles, and FHC between infants of different ages. Pearson correlation analysis was used to determine the correlation between age and α/β angles and FHC and between FHC and α/β angles. Correlation coefficients of |r| ≥ 0.8 indicate strong correlation; 0.5 ≤ |*r*| < 0.8 indicate moderate correlation; 0.3 ≤ |*r*| < 0.5 indicate weak correlation; and |*r*| < 0.3 indicate no correlation. Differences with *p* < 0.05 were considered statistically significant.

## Results

A total of 3,088 infants were included in the study. There were 2,083 cases of both hips diagnosed as Graf type I and 1,005 cases diagnosed as Graf type IIa (unilateral or bilateral) at their initial examination. None of the type IIa hips was treated, of which 984 cases developed into type I. Twenty one cases were diagnosed as type IIb (12 left, 7 right, and 2 bilateral) on re-examination after 3 months of age and were therefore excluded from this study. A total of 3,067 cases (6,134 hips) were finally included in the analysis (1,164 males and 1,903 females). The mean age was 77 days with the range of 1–180 days.

When comparing by side, the α angle and FHC were slightly smaller on the left side than on the right side while the β angle was slightly larger on the left side than on the right side, (*p* < 0.001). These findings indicated that hip development on the left side slightly lagged behind the right side (Table 1). When comparing by sex, the α angle on both sides were significantly smaller in females than in males (*p* < 0.001), whereas the β angle on the left side was slightly larger in females than in males (*p* = 0.014). There was no statistically significant difference in the β angle on the right side between the two sexes (*p* = 0.150). FHC on both sides was slightly smaller in females than in males (*p* < 0.001), indicating that hip development in females, particularly on the left side, slightly lagged behind that in males (Table 2).

**Table 1:** Comparison of α angle, β angle, and femoral head coverage between left hips and right hips in normal infants.

**Measurements**	**Left (3,067 hips)**	**Right (3,067 hips)**	***T-*valu*e***	***P-*value**
α angle (°)	62.4 ± 3.6	63.2 ± 3.5	−13.1	<0.001
β angle (°)	55.6 ± 4.5	54.8 ± 4.5	10.8	<0.001
FHC (%)	54.2 ± 4.6	54.8 ± 4.2	−8.4	<0.001

*The P-value was calculated by using Paired sample student's t-test. FHC, femoral head coverage*.

**Table 2:** Comparison of α angle, β angle, and femoral head coverage of the hip between sexes in normal infants.

**Measurements**	**Side**	**Male (1,164 cases)**	**Female (1,903 cases)**	***T-*value**	***P-*value**
α angle (°)	Left	63.3 ± 3.6	61.8 ± 3.6	11.5	<0.001
	Right	64.0 ± 3.5	62.7 ± 3.4	10.0	<0.001
β angle (°)	Left	55.3 ± 4.5	55.7 ± 4.5	−2.5	0.014
	Right	54.7 ± 4.6	54.9 ± 4.4	−1.4	0.150
FHC (%)	Left	54.9 ± 4.4	53.7 ± 4.6	6.9	<0.001
	Right	55.3 ± 4.2	54.5 ± 4.2	5.5	<0.001

*The P-value was calculated by using Independent sample student's t-test. FHC, femoral head coverage*.

**Figure 2:**
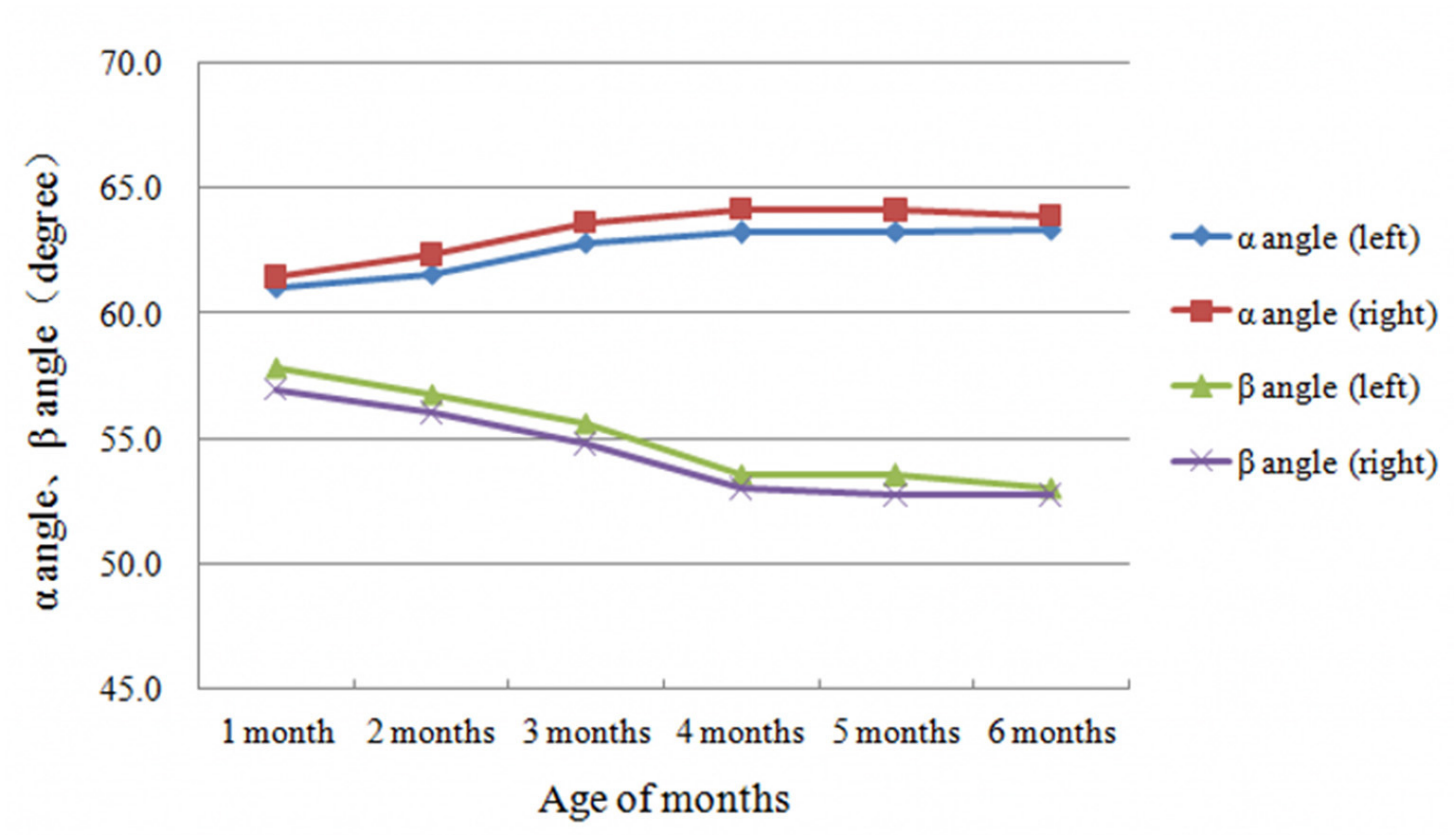
Laterality characteristics of α and β angles development with age of months.

**Figure 3:**
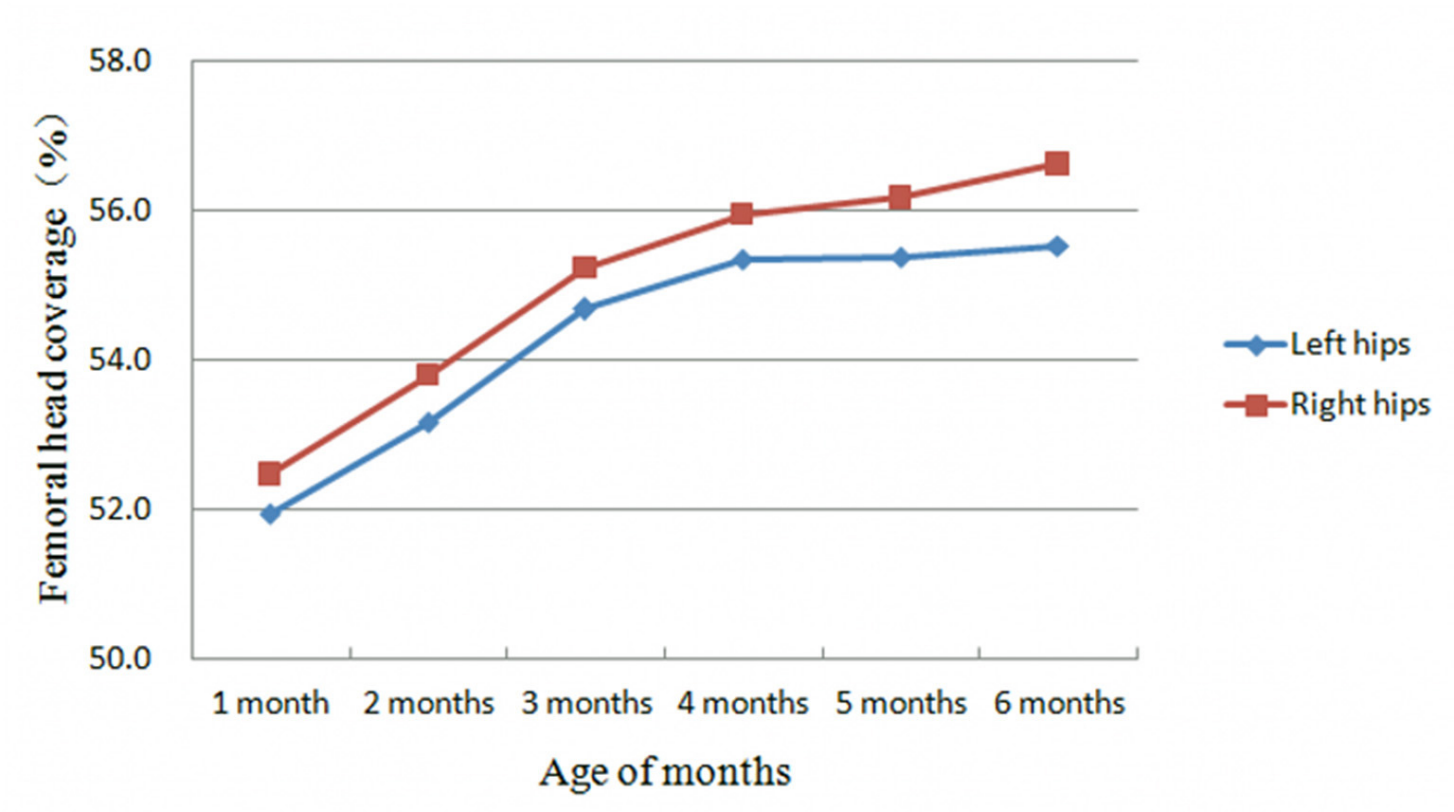
Development trend of femoral head coverage with age of months.

The α and β angles, and FHC exhibited similar characteristic at different months of age. In the first 3 months after birth, the α angle and FHC tended to increase each month while the β angle tended to decrease each month (Tables 3–**5**, Figures 2, 3). In contrast, between 4 and 6 months after birth, the α and β angles, and FHC were stabilized at relatively constant levels and did not change with age (Tables 3–**5**, Figures 2, 3).

**Table 3:** Comparison of α angle, β angle, and femoral head coverage of the hips among ages of months in normal infants.

**Age (months)**	***N* (hips)**	**α angle (°)**	**β angle (°)**	**FHC (%)**
1	500	61.3 ± 4.1	57.4 ± 5.1	52.2 ± 5.0
2	2,020	61.9 ± 3.6	56.4 ± 4.6	53.5 ± 4.4
3	1,778	63.2 ± 3.5	55.2 ± 4.5	55.0 ± 4.0
4	1,190	63.7 ± 3.2	53.3 ± 3.3	55.7 ± 4.2
5	402	63.7 ± 3.1	53.1 ± 3.0	55.8 ± 3.8
6	244	63.6 ± 3.1	52.9 ± 3.2	56.1 ± 4.0
*P-*value		<0.001	<0.001	<0.001

*The P-value was calculated by using ANOVA test. FHC, femoral head coverage*.

Pearson correlation analysis showed that α angle, β angle, and FHC exhibited weak or no correlation with age (*r* = 0.224, −0.305, and 0.254, respectively, *p* < 0.001). FHC exhibited moderate positive correlation with α angle (*r* = 0.594, *p* < 0.001) and weak negative correlation with β angle (*r* = −0.304, *p* < 0.001).

## Discussion

This is one of the first comprehensive studies of hip development in Chinese infants. Previously, we observed the developmental characteristics of acetabular morphology in Chinese children using magnetic resonance and found that infancy is a period of rapid decline in bony and cartilaginous acetabular indices (10). However, detailed monthly data during infancy is lacking. The present study supplements these previous results by detailing the morphological pattern of the hip joint development in 3,067 normal Chinese infants under 6 months of age. With respect to sample selection, the possible effects of high risk factors associated with DDH, such as prematurity, family history, breech delivery, and oligohydramnios, on hip development were excluded. In addition, type IIa hips with persistent immaturity were excluded so that the included samples were as close to the population of normally developing infants as possible.

The results of the present study showed that during the first 3 months of life, the α angle and FHC exhibit a monthly increase while the β angle exhibits a monthly decrease (Figures 2, 3). The differences in all parameters were statistically significant among different age groups (Tables 4, 5). However, between 4 and 6 months of life, instead of changing with age, the α and β angles, and FHC stabilized at a relatively constant level (Figures 2, 3). These findings are consistent with data from Cheng et al. (9) and Riad et al. (11), in which longitudinal results were obtained only at birth, 6 and 12 weeks. All of these studies indicate that the first 3 months of life is a period of rapid development of the hip joint, when both bony and cartilaginous acetabular roof margins mature gradually. This observation suggests that the first 3 months of life is a critical period for hip joint plasticity, and that DDH is likely to return to normal development within its inherent plasticity if properly treated during this period. In fact, this hypothesis that has been validated by many clinical studies (12–14). On the other hand, our study also confirms the importance of early screening for DDH in the neonatal period. Conversely, if normal development of the acetabulum is disturbed during this period by various environmental factors, such as straight-leg swaddling, irreversible acetabular dysplasia may result. In previous animal studies, earlier onset and longer duration of postnatal straight-leg swaddling resulted in more severe acetabular dysplasia (15). Understanding these developmental patterns of the hip joint is important for the selection of treatment modalities and determination of prognosis for DDH. For example, the identification of Graf type IIa and IIb hips by the age of 3 months fully takes into account the developmental pattern of the hip joint based on monthly age. Type IIa hips can develop normally without any intervention by 3 months of age in over 90% of cases (16, 17), confirming the sufficient developmental potential of the hip joint during this period.

**Table 4:** Multiple significance comparisons of α angle and β angle over age of months in normal infants.

		**β angle by age (months)**					
		**1**	**2**	**3**	**4**	**5**	**6**
α angle by age (months)	1	-	0.000	0.000	0.000	0.000	0.000
	2	0.000	-	0.000	0.000	0.000	0.000
	3	0.000	0.000	-	0.000	0.000	0.000
	4	0.000	0.000	0.000	-	0.534	0.196
	5	0.000	0.000	0.017	0.898	-	0.498
	6	0.000	0.000	0.091	0.736	0.841	-

*The data in the table represent the P-values calculated by analysis of variance test. The values in the bottom left semi-part of the table are the multiple comparisons of α angle by age, and the right upper semi-part is the multiple comparisons of β angle by age*.

**Table 5:** Multiple significance comparisons of femoral head coverage over age of months in normal infants.

	**Femoral head coverage by age (months)**					
	**1**	**2**	**3**	**4**	**5**	**6**
1	-					
2	0.000	-				
3	0.000	0.000	-			
4	0.000	0.000	0.000	-		
5	0.000	0.000	0.000	0.628	-	
6	0.000	0.000	0.000	0.165	0.391	-

*The data in the table represent the P-values calculated by analysis of variance test*.

Interestingly, the present study reveals that there were significant laterality and gender differences in α and β angles, and FHC. The α angle on the left side was consistently smaller than that on the right side in all age groups, whereas the β angle on the left side was consistently larger than on the right side. This difference did not exceed 1° but was statistically significant (Table 1, Figure 2). Due to the relatively large sample size of the present study, the effect of systematic error was excluded to some extent. Cheng et al. (9) performed ultrasound measurements of the hip joints in normal infants between 0 and 6 months of age and also confirmed that the laterality differences in β angle. However, no significant difference in α angle between the left and right sides was observed. Our results suggest that the development of the bony and cartilaginous acetabular roof of the hip joint in normal infants inherently lags slightly on the left side compared to the right. In addition, these parameters differed significantly between the sexes, with the α angle on both sides being significantly smaller in females than in males (about 1.5°). The FHC on both sides was also slightly smaller in females than in males, with a difference of about 1%. However, FHC difference was not clinically significant. While the β angle on the left side was slightly larger in females than in males, there was no statistically significant difference in the β angle on the right side between the two sexes. These results suggest that the development of the hip joint in normal infants inherently lags slightly in females compared to males, with the left hip lagging more significantly. This is consistent with the clinical phenomenon that DDH is more common in females and more often involves the left hip. However, the reasons for such gender and laterality differences remain unclear and require further in-depth investigation.

Correlation analysis showed that α and β angles, and FHC exhibited only weak or no correlation with monthly age, with a maximum correlation coefficient |r| of 0.305 (*p* < 0.001), this finding may be explained by various factors. First, the change in each indicator was statistically significant in the first 3 months but its magnitude is small (Table 3). Second, the three parameters mentioned above plateaued between 4 and 6 months and did not vary with monthly age, resulting in a lack of significant correlation with monthly age. However, α angle was moderately positively correlated with FHC (*r* = 0.594, *p* < 0.001), which was consistent with findings by Gunay et al. (18) and Czubak et al. (19) (*r* = 0.668, *p* = 0.001). The α angle represents the degree of development of the bony acetabular roof, whereas FHC reflects the relationship between the bony acetabular roof in relation to the diameter of the femoral head. The positive correlation between the two indicates that the development of the bony acetabulum and the femoral head during this period of 0–6 months is approximately coordinated (9). Understanding this pattern can help with identifying hip abnormalities under 6 months of age and monitoring the developmental process after DDH treatment (20, 21).

Although the present study elucidated the normal values and extents of variability in α and β angles, and FHC at each month during the first 6 months of life, its data differed from those of similar studies. This discrepancy results from variations in ultrasound equipment used at each study center, the position of the infant during the examination, the orientation of the ultrasound probe, and the time of measurement (22, 23). Nevertheless, this study provides new insights on the developmental pattern of the hip joint in normal Chinese infants under 6 months of age and serves as an important reference for clinical diagnosis of DDH.

The present study has some limitations. First, it was a retrospective study with inevitable selection bias and measurement errors. Second, no further distinction was made between Graf type IIa^+^ and IIa^−^ hips. Long-term longitudinal observation was also not performed. Therefore, the possibility of long-term persistent dysplasia in the selected participants cannot be excluded. Third, intra- and inter-observer consistency analysis of measurement results was not performed, which affects the reliability of the measured data to some extent. Finally, the study sample represents a spontaneous outpatient screening population, instead of a general random population. The sampling method was not used to select the infants with the hips of Graf I and IIa, which might increase the selection bias. These limitations should be remedied in the further prospective studies.

## Conclusion

This study elucidated the developmental patterns of α angle, β angle, and FHC in normal Chinese infants under 6 months of age. The first 3 months after birth is a period of rapid development of the hip joint, whereas 4–6 months of age is a plateau period. In addition, there are laterality and gender differences in these Graf-related parameters. The development of the female hip and the left hip inherently lags slightly behind that of the male and right hip during this period. These findings may explain the clinical observation that DDH is more common in females and on the left side. Knowledge of these age-related developmental characteristics of the hip is essential for clinicians to properly assess the morphology of the hip joint in infants.

## Data Availability Statement

The original contributions presented in the study are included in the article/supplementary material, further inquiries can be directed to the corresponding author.

## Ethics Statement

The studies involving human participants were reviewed and approved by Institutional Review Board of Shengjing Hospital, China Medical University. Written informed consent to participate in this study was provided by the participants' legal guardian/next of kin.

## Author Contributions

BL conducted data collection and analysis, drafted the manuscript. XH completed the data analysis and manuscript revision. SG performed ultrasound measurement and data analysis. LL was in charge of study design, project administration, fund acquisition, and manuscript revision. All authors contributed to the article and approved the submitted version.

## Funding

The study was funded by the National Natural Science Foundation of China (81772296).

## Conflict of Interest

The authors declare that the research was conducted in the absence of any commercial or financial relationships that could be construed as a potential conflict of interest.

## Publisher's Note

All claims expressed in this article are solely those of the authors and do not necessarily represent those of their affiliated organizations, or those of the publisher, the editors and the reviewers. Any product that may be evaluated in this article, or claim that may be made by its manufacturer, is not guaranteed or endorsed by the publisher.
